# Photophysical Properties of Protoporphyrin IX, Pyropheophorbide-a, and Photofrin^®^ in Different Conditions

**DOI:** 10.3390/ph14020138

**Published:** 2021-02-09

**Authors:** Bauyrzhan Myrzakhmetov, Philippe Arnoux, Serge Mordon, Samir Acherar, Irina Tsoy, Céline Frochot

**Affiliations:** 1 LRGP UMR 7274, CNRS, University of Lorraine, 54000 Nancy, France; bauyrzhanmyrzakhmetov86@gmail.com (B.M.); philippe.arnoux@univ-lorraine.fr (P.A.); 2 Department of Chemistry and Chemical Technology, M.Kh. Dulaty Taraz Regional University, Taraz 080012, Kazakhstan; tsoyirinagen@mail.ru; 3 ONCO-THAI U1189, INSERM, CHU Lille, University of Lille, 59000 Lille, France; serge.mordon@inserm.fr; 4 LCPM UMR 7375, CNRS, University of Lorraine, 54000 Nancy, France; samir.acherar@univ-lorraine.fr

**Keywords:** photodynamic therapy, protoporphyrin IX, pyropheophorbide-a, Photofrin^®^, absorption, fluorescence, singlet oxygen

## Abstract

Photodynamic therapy (PDT) is an innovative treatment of malignant or diseased tissues. The effectiveness of PDT depends on light dosimetry, oxygen availability, and properties of the photosensitizer (PS). Depending on the medium, photophysical properties of the PS can change leading to increase or decrease in fluorescence emission and formation of reactive oxygen species (ROS) especially singlet oxygen (^1^O_2_). In this study, the influence of solvent polarity, viscosity, concentration, temperature, and pH medium on the photophysical properties of protoporphyrin IX, pyropheophorbide-a, and Photofrin^®^ were investigated by UV-visible absorption, fluorescence emission, singlet oxygen emission, and time-resolved fluorescence spectroscopies.

## 1. Introduction

Photodynamic therapy (PDT) is a targeted technique for the treatment of malignant or diseased tissues that relies on three non-toxic elements—a light-activated drug (photosensitizer, PS), light, and molecular oxygen. Illumination of the PS induces the production of the triplet excited state ^3^PS* which is able to transfer protons, electrons, or energy, leading to the formation of reactive oxygen species (ROS). ROS cause apoptosis or necrosis of tumor cells by photochemical oxidation [1,2,3].

A PS should ideally possess some valuable properties including (i) absorption peak in the near-infrared (NIR) region (700–1000 nm) of the UV-visible spectrum that provides enough penetration of light into deep tissues and energy to excite molecular oxygen to its singlet state efficiently, (ii) minimal skin photosensitivity, (iii) no dark toxicity, (iv) selective uptake by cancer tissues, thereby enabling the decrease of side effects [4,5], and (v) fast elimination.

The self-assembly or aggregation of PS in aqueous environments can be caused by different reasons and is favored for amphiphilic PSs showing a negligible PDT activity due to the emission reduction of the ^3^PS* state in aggregated form [6,7]. The physico-chemical properties of aggregates differ from those of monomers. They exhibit a broadened Soret band and red-shifted Q bands in the UV-visible absorption spectra, low fluorescence intensity and lifetime [8,9,10,11,12,13], and low singlet oxygen (^1^O_2_) production.

The hematoporphyrin derivative (HPD) and its purified form Photofrin^®^ (PF) were the first used PSs in PDT and PF was approved for the treatment of solid tumors [14,15,16,17]. PF was also indicated as a specific and selective radiosensitizing agent by several in vitro and in vivo studies [18,19,20,21,22]. PF is being used for the treatment of esophageal and non-small-cell lung and pancreatic cancers as well as a possible therapy against Karposi’s sarcoma and brain, breast, skin, and bladder cancers [23].

The selective accumulation of protoporphyrin IX (PpIX) in the tumor cells following administration of 5-aminolevulinic acid (5-ALA) has made this PS precursor very popular for skin cancer PDT and fluorescent diagnostics of tumor tissues [24,25]. Topical, oral, or intravenous administration of 5-ALA prodrug in excess leading to the formation and accumulation of PpIX in vivo [26] is used by dermatologists to treat several malignant neoplasms of the skin, such as Bowen disease or actinic keratosis [27].

Pyropheophorbide-a (PPa) is a natural second-generation bacteriochlorin PS which presents a significant absorption in the far-red spectral region and high ^1^O_2_ formation upon light illumination, suggesting it for PDT [28,29].

This study aimed to explore the different parameters (i.e., solvent polarity, concentration, temperature, and pH medium) that influence the photophysical properties (absorption, fluorescence emission, and ^1^O_2_ formation) of the three PSs (PpIX, PPa, and PF).

## 2. Results and Discussion

**Figure 1:**
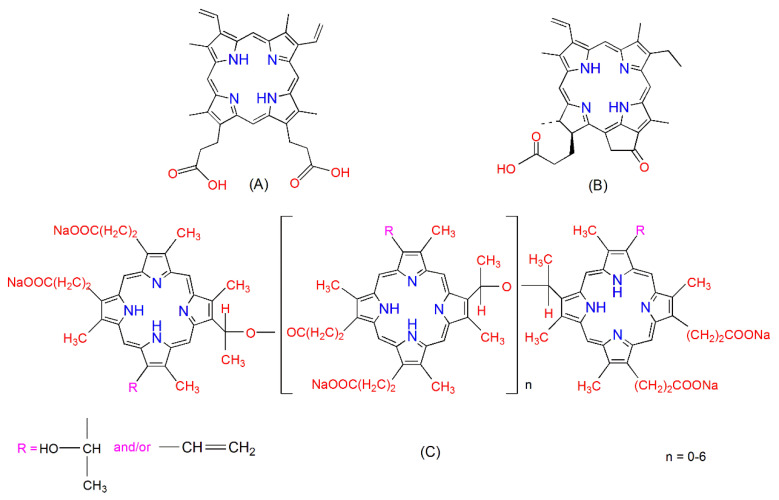
Chemical structures of protoporphyrin IX (PpIX) (**A**), pyropheophorbide-a (PPa) (**B**), and Photofrin^® (^PF) (**C**).

Chemical structures of PpIX, PPa, and PF are shown in Figure 1. PpIX has two ionizable propionate groups and a hydrophobic ring core, which gives it amphiphilic properties leading to an aggregation through π–π stacking interaction and vesicle formation [30]. PPa has only one propionate group and a hydrophobic ring core, it can also aggregate in aqueous solutions [31]. PF is composed of monomers, dimers, and some very large oligomers [32].

### 2.1. Influence of the Solvent

**Figure 2:**
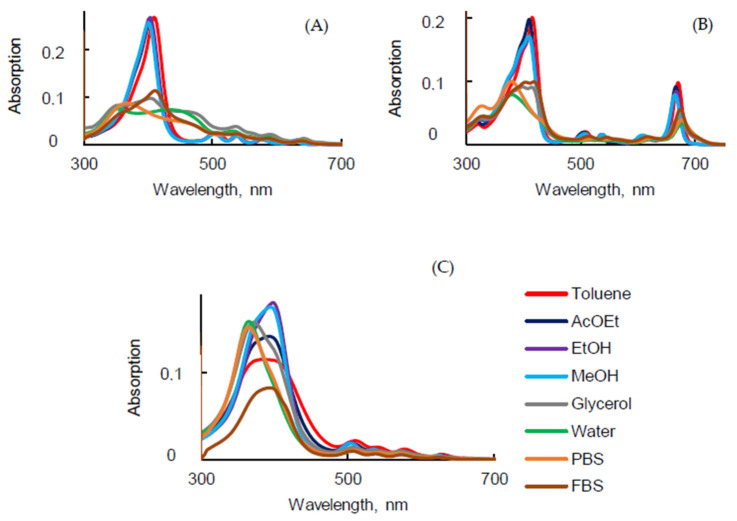
UV-visible absorption spectra of PpIX (**A**), PPa (**B**) and PF (**C**) in different solvents (*c* = 1.87 μM).

The structure of the molecule, ionic strength, pH, and temperature should play a main role in the photophysical properties [33]. UV-visible absorption spectra of PSs presented in Figure 2, were recorded in different solvents. ET(30) is a solvent polarity parameter that characterized the polarity of the different solvents. The bigger the ET(30) value, the more solvent polarity it is associated with.

As expected, UV-visible absorption spectra of PpIX (Figure 2A) exhibited an intense Soret band centered at around 406 nm and four weaker Q bands in the visible range in toluene, ethyl acetate (AcOEt), ethanol (EtOH), and methanol (MeOH). These similar spectra are typical of monomeric PpIX. Nevertheless, in glycerol, water, phosphate-buffered solution (PBS), and fetal bovine serum (FBS), the Soret band was split into two bands [34]. This can be explained by the fact that PpIX is aggregated in aqueous solutions. In polar solvents the QI band was red-shifted compared to the QI band in less polar solvents (629 nm and 641 nm in EtOH and PBS, respectively) (Table 1), and the intensity decreased drastically. In the literature, it is often claimed that PpIX should be excited in vitro or in vivo at 630 nm. This wavelength of excitation is based on the absorption spectrum in EtOH. As it can be seen in Table 1 in water, PBS, and FBS, the QI band was located at 641, 641, and 640 nm, respectively.

The UV-visible absorption spectra of PPa in various solvents exhibited a Soret band and four Q bands in the spectral range 300–700 nm (Figure 2B and Table 1). The Soret band in non-aqueous solution was located between 409 nm and 415 nm whereas it was blue-shifted to 380 nm in water and PBS and 405 nm in FBS. QIV, QIII, QII, and QI were red-shifted from, respectively, 510 nm to 526 nm, 539 nm to 558 nm, 612 nm to 630 nm, and 671 nm to 677 nm, from toluene to PBS. The UV-visible absorption spectra of PPa showed a broad Soret band in glycerol, water, PBS, and FBS due to the formation of aggregates. Interestingly, all shifts of PPa in glycerol and FBS showed close values.

UV-visible absorption spectra of PF in the different solvents were less impacted by the change of the polarity than PpIX and PPa. The shape of the Soret band was broader in toluene, AcOEt and it became intense when the polarity of solvent increased. The Soret band was blue-shifted by 23 nm from toluene to PBS. The positions of QI and QII bands in water and PBS were blue-shifted by 12 nm, respectively, compared to toluene (Figure 2C). The maxima of the absorption bands are presented in Table 1. In FBS, due to the presence of proteins (30–45 g·L^−1^), the behavior was different to than in water. The absorption spectra in FBS were similar to those in toluene.

On the basis of the UV-visible absorption spectra of PSs, molar extinction coefficients (ε) were calculated for all observed bands in all solvents. For PpIX and PPa, there was a single abrupt jump of ε (for the Soret band) when moving from toluene, AcOEt, EtOH, or MeOH into glycerol, water, or PBS. That was not observed for PF due to the fact that PF is a mixture of different compounds that do not all behave in the same way (Table 2). The high value of ε for the QI band of PPa is interesting for PDT applications. ε for the QI band of PPa was 3.5 times higher than the one of PpIX and 16.5 times higher than the one of PF [35].

**Figure 3:**
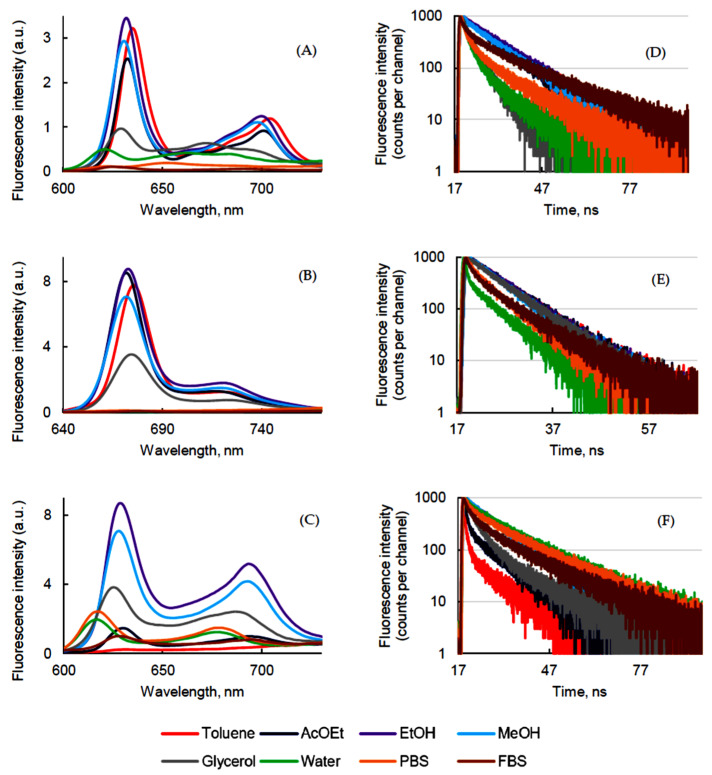
Fluorescence emission and decay (λ_exc_ = 408 nm) of PpIX (**A**,**D**), PPa (**B**,**E**), and PF (**C**,**F**) in different solvents (*c* = 1.87 μM).

Fluorescence emission spectra presented in Figure 3 were recorded in different solvents at room temperature and at a concentration of 1.87 μM.

PpIX was excited at 400 nm. The highest was the polarity of the solvent and the blue-shift of the two fluorescence emission bands in agreement with the UV-visible absorption spectra (Figure 3A). All the maximum wavelengths are in the supporting information. Moreover, fluorescence intensity decreased with increase of the polarity of the solvent due to the lack of solubility of PpIX in aqueous media.

PPa was excited at 415 nm. The fluorescence emission spectra presented two bands and they were blue-shifted in polar solvents in accordance with the blue shift observed in the UV-visible absorption spectra. In water, PBS, and FBS at this concentration, the fluorescence intensity was very weak (Figure 3B), which could be explained by the aggregation [31].

PF was excited at 400 nm and a different behavior was observed in fluorescence emission spectra. Very weak maximum emission peaks at 633 nm and 696 nm were observed in toluene. The PF cores might have been in their highly quenched state, which did not generate fluorescence. The fluorescence intensity increased around 10 times in EtOH, and decreased again in polar solvents (Figure 3C). The emission bands of PF in water and PBS were blue-shifted for 15 ± 2 nm (Table S1) like the UV-visible absorption spectra indicating highly ordered aggregated structures [36]. In FBS, the fluorescence spectrum was similar to the one in non-polar solvent.

The fluorescence quantum yield (Φ_f_) of PpIX was evaluated to be higher in less polar solvents than in water, PBS, and FBS (Table 3). This is in good agreement with the fact that PpIX tends to aggregate in aqueous media [37]. Among the three PSs, PPa presented the best Φ_f_ which was 0.39 in toluene and EtOH. Φ_f_ of PF was low (below 0.1) and the highest was obtained in EtOH and MeOH, possibly due to a better solubilization (Table 3).

The fluorescence lifetime (τ*_f_*) of PSs was measured by time-resolved fluorescence after excitation at 408 nm. An exponential decay was fitted with an R^2^ ≈ 1.000. A bi-exponential decay of PpIX in polar solvents confirmed the presence of two populations—monomers (long decay) and aggregates (short decay). PpIX exhibited mono-exponential decay (Figure 3D) in non-polar solvents, though the monomer–aggregate equilibrium was observed in glycerol, water, PBS, and FBS solutions: τ*_f_* values of 10.3–15.9 ns and 2.5–3.0 ns for PpIX monomer and aggregates, respectively (Table 4).

As it is known that aggregates reduce the inter-system crossing (ISC) transition from ^1^PS* to ^3^PS* and τ*_f_* of aggregated PPa was shorter. PPa exhibited mono-exponential and bi-exponential decay in toluene, AcOEt, EtOH, MeOH, glycerol, and in water, PBS, and FBS, respectively (Figure 3E), confirming the presence of two forms—monomers (long decay) and aggregates (short decay). The τ*_f_* value of PPa was between 6.1 to 7.5 ns for monomers and 0.3 to 2.1 ns for aggregates (Table 4).

The solution of PF in toluene, EtOH, and MeOH exhibited mono-exponential decay with τ*_f_* values of 8.7, 10.8, and 10.2 ns, respectively, which was in good agreement with literature values [38,39], and two decays in the other solvents (Figure 3F). Once again, the short decay corresponded to the aggregated parts with lifetime of 2.4, 3.0, 3.4, 2.2, and 3.2 ns in AcOEt, glycerol, water, PBS, and FBS, respectively, since there was a longer decay for monomers (Table 4).

**Figure 4:**
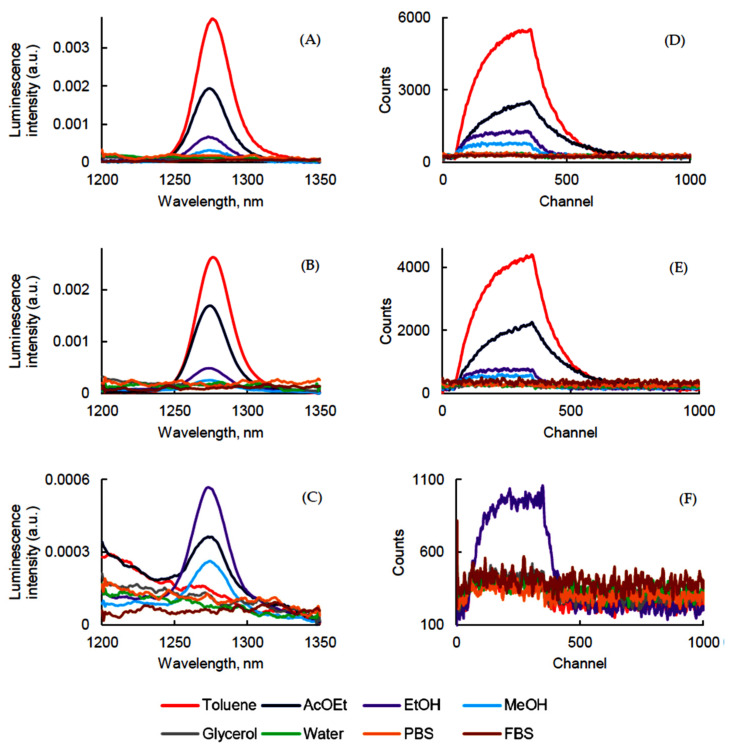
^1^O_2_ luminescence emission and decay (λ_exc_ = 400 nm for PpIX and PF and 415 nm for PPa) of PpIX (**A**,**D**), PPa (**B**,**E**), and PF (**C**,**F**) in different solvents (*c* = 1.87 μM).

The ^1^O_2_ production of PSs in different solvents was carried out and ^1^O_2_ emission was detected at 1270 ± 5 nm after excitation at 400 nm for PpIX, PF, and 415 nm for PPa (Figure 4).

**Figure 5:**
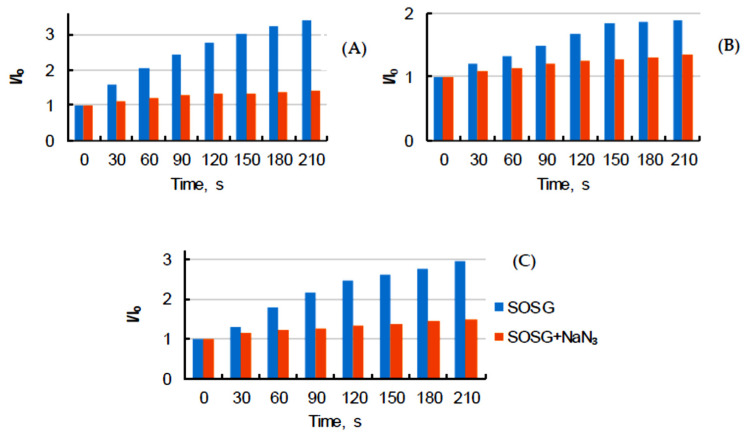
Comparison of fluorescence emission intensity of Singlet Oxygen Sensor Green (SOSG) and SOSG + NaN_3_ in D_2_O for ^1^O_2_ detection after excitation of PpIX (**A**), PPa (**B**), and PF (**C**) (λ_exc_ = 400 nm for PS and 495 nm for SOSG) (*c* = 3.1 μM).

As expected, it was not possible to determine the Φ_Δ_ of PpIX in aqueous solutions due to possible aggregation of the PS, but it generated ^1^O_2_ very efficiently in toluene, AcOEt, EtOH, and MeOH (Figure 4A). The same observation could be made with PPa. In our conditions we could not detect ^1^O_2_ emission in glycerol, water, FBS, or PBS (Figure 4B). On the contrary, PF (Figure 4C) generated ^1^O_2_ in EtOH, AcOEt, and MeOH (Table 5. The detection of ^1^O_2_ was performed in D_2_O, since the τ_Δ_ value is higher than in H_2_O. Indeed, solvents with high vibrational frequencies are more able to quench ^1^O_2_ [40]. However, no emission could be detected for PpIX and PPa whereas a Φ_Δ_ of 0.15 was obtained for PF. Additionally, ^1^O_2_ generation from PpIX, PPa, and PF in D_2_O was monitored by using the most common fluorescence probe Singlet Oxygen Sensor Green (SOSG), which is not sensitive to hydroxyl radicals or superoxide. It clearly appeared that fluorescence emission intensity of SOSG increased during the time due to the production of ^1^O_2_ after excitation of PpIX, PPa, and PF. Sodium azide quenched ^1^O_2_ very efficiently and fluorescence emission intensity of SOSG in the presence of quencher decreased (Figure 5).

^1^O_2_ lifetime (τ_Δ_) was determined. In solution, τ_Δ_ is governed by solvent deactivation through electronic-vibrational energy transfer [41]. If no reaction happens between ^1^O_2_ and PS, τ_Δ_ value should be the same for the three PSs in each solvent. What we can observe is in good relation with the literature data (Table 6).

### 2.2. Influence of the Medium Viscosity

**Figure 6:**
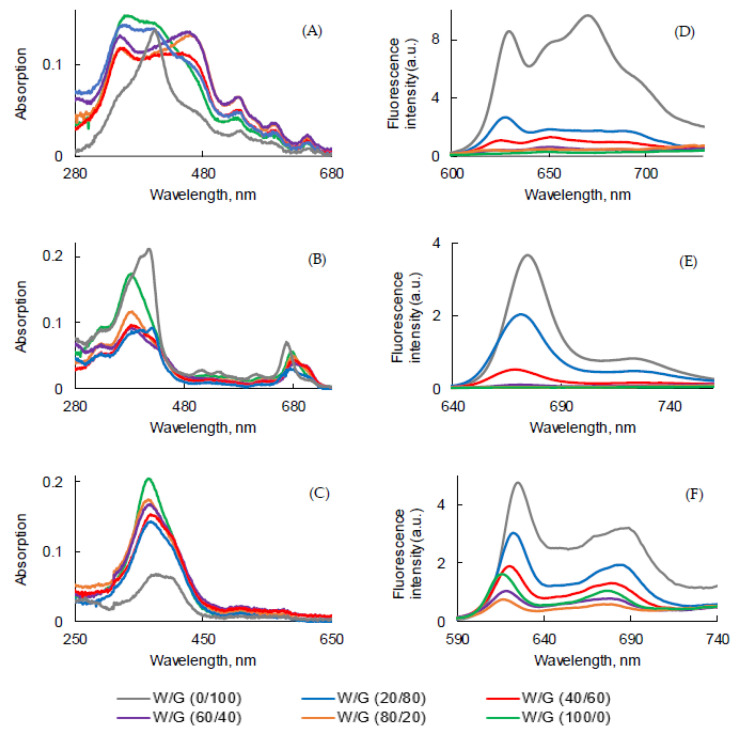
UV-visible absorption and fluorescence emission spectra ((λ_exc_ = 400 nm for PpIX, PF and 415 nm for PPa) of PpIX (**A**,**D**), PPa (**B**,**E**), and PF (**C**,**F**) in water/glycerol (W/G) mixtures (*c* = 3.1 μM) at room temperature.

To evaluate the influence of the viscosity on the photophysical properties, a water/glycerol (W/G) mixture at various ratios was used. The higher the glycerol concentration, the higher the viscosity of the medium. The UV-visible absorption and fluorescence emission spectra of all PSs are shown in Figure 6.

For the three PSs, fluorescence emission decreased with addition of water. The highest was the viscosity and the lowest was the non-radiative decay.

The Soret band became larger and split in the solutions of PpIX with high concentrations of water, but the maximum wavelengths of four Q bands were not affected. A thin Soret band at 406 nm was only observed in 100% glycerol. This might be due to the fact that with the increase of the viscosity, the movement of the molecules was reduced and the formation of aggregates decreased or just due to the fact that aggregation occurred in water (Figure 6A).

UV-visible absorption spectra of PPa in water and in the mixture of water/glycerol showed a blue-shifted Soret band and weak, red-shifted Q bands. These band shifts might have been a result of the viscosity, which reduced the molecule’s mobility for aggregate formation (Figure 6B).

A totally different behavior was observed for PF. The intensity of the Soret band of PF decreased by increasing the viscosity of the medium and the Soret band became wider in 100% glycerol with a red-shift of the maximum of absorption (Figure 6C).

**Figure 7:**
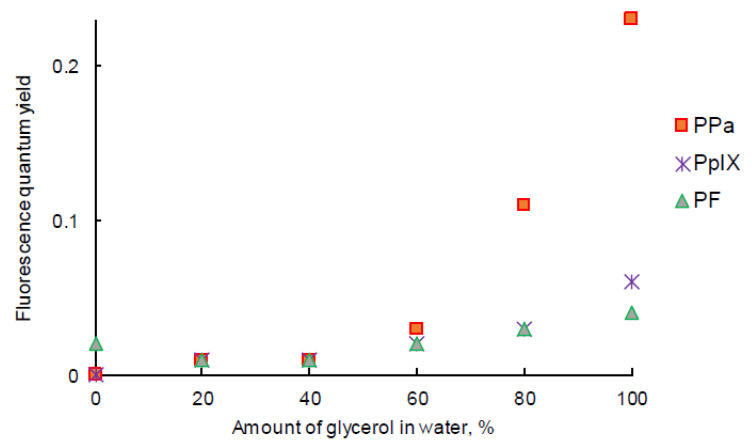
Fluorescence quantum yield of PpIX, PPa, and PF in the water/glycerol (W/G) mixture (*c* = 3.1 μM).

The intensity of fluorescence emission of PpIX in the W/G mixture increased with the viscosity of the medium (Figure 6D). Φ_f_ value of PpIX in the W/G mixture increased in highly viscous media due to the fact that the formation of aggregates was less important. PPa in glycerol showed two emission bands located at 675 nm and 724 nm (Figure 6E). The fluorescence emission intensity increased with the viscosity of medium. The viscous medium might prevent non radiative deactivation. The fluorescence emission intensity of PF also increased with the viscosity of the medium (except in water) and was red-shifted (Figure 6F) for 10 nm. It is interesting to note that for PF when the viscosity increased, fluorescence emission increased but absorption decreased. The highest Φ_f_ value for all PSs was calculated for the solution in glycerol (Figure 7).

Fluorescence emission decays presented in Figure S1 were measured in the different media. τ*_f_* were evaluated and are presented in Table 7. In all mixtures two lifetimes were detected, probably because of the presence of both monomers and aggregates. The τ*_f_* value of PpIX increased with the viscosity (Figure S1A). The solution of PPa in W/G (100/0, 80/20, and 60/40) ratio exhibited two decays, but starting at a ratio of 40/60 showed mono-exponential decay and τ*_f_* increased in line with the medium viscosity (Figure S1B). The solution of PF exhibited bi-exponential decay (Figure S1C) in all W/G mixtures.

Unfortunately, no correlation could be established between the fraction of monomers/aggregates and the viscosity of the medium. One reason might be that the polarity of the medium also changes when different amounts of glycerol and water are mixed. Therefore, the changes observed in W/G mixtures cannot only be attributed to the solution viscosity.

### 2.3. Influence of the Concentration

**Figure 8:**
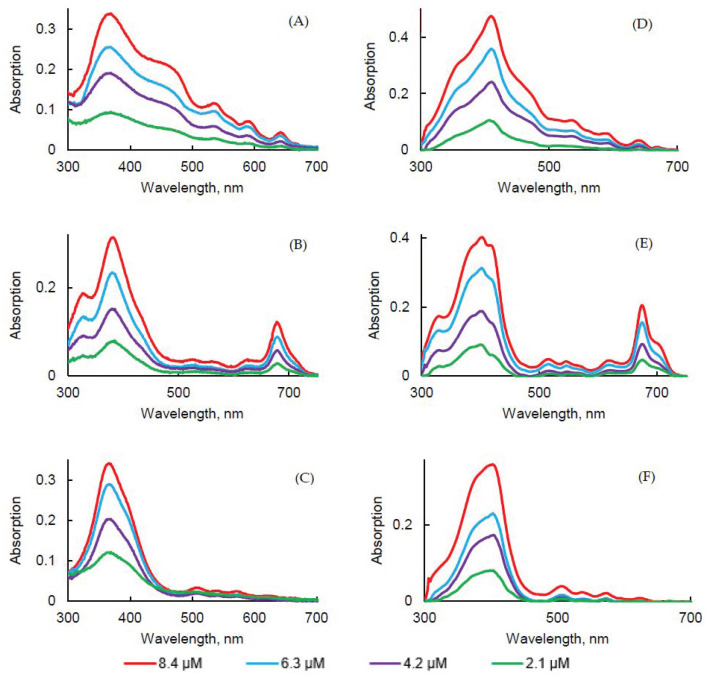
UV-visible absorption spectra of PpIX (**A**,**D**), PPa (**B**,**E**), and PF (**C**,**F**) in PBS (**A**–**C**) and FBS (**D**–**F**) at different concentrations.

The influence of the concentration of PSs in PBS and FBS on photophysical properties was evaluated. As expected, the increase in concentration induced an increase in intensity, but no change in the absorption band maximum wavelength was observed in this concentration range for all PSs (Figure 8A–C).

**Figure 9:**
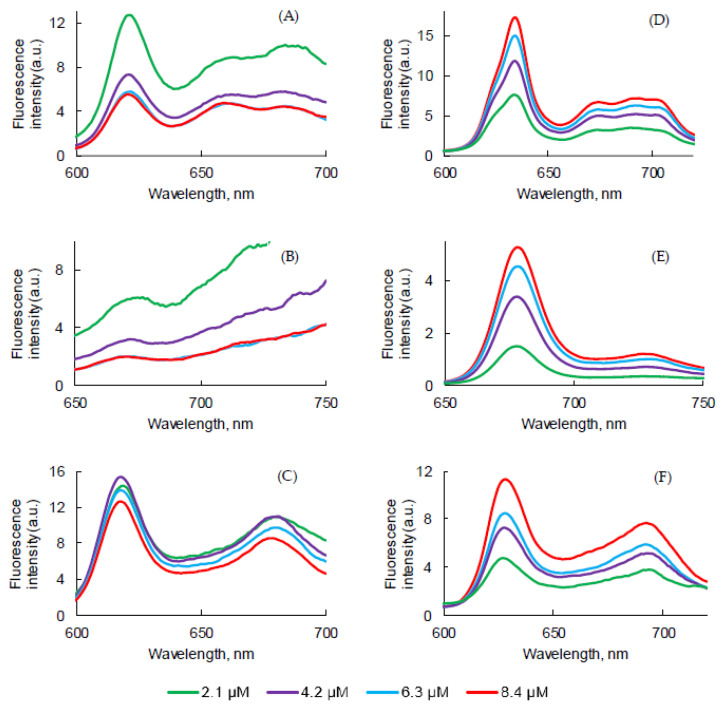
Fluorescence emission spectra (λ_exc_ = 400 nm for PpIX, PF and 415 nm for PPa) of PpIX (**A**,**D**), PPa (**B**,**E**), and PF (**C**,**F**) in PBS (**A**–**C**) and FBS (**D**–**F**) at different concentrations.

The concentration increase led to a decrease of the fluorescence emission intensity for all PSs. Aggregation was higher in concentrated solutions (Figure 9D–F). The higher was the concentration, the lower was the fluorescence. Φ_f_ of all PSs in PBS at different concentrations were measured and were all less than 1%. However, the results obtained in FBS turned out to be the opposite in comparison with PBS. As the concentration of all PSs increased, fluorescence emission of all PSs increased. The aggregation process might be lower in FBS than in PBS due to the interaction with the proteins.

Fluorescence decays were recorded (Figure S2A) and τ*_f_* were evaluated (Table 8). PpIX in PBS or FBS at different concentrations exhibited bi-exponential decay. The longest τ*_f_* likely corresponded to the monomer decay time and the shorter lifetime was likely due to aggregates’ decay time. PpIX in PBS at different concentrations exhibited bi-exponential decay. We could observe a slight increase of the ratio aggregate/monomers with the concentration increase in PBS but not in FBS. For PPa, only one population was observed in PBS between 5.6 and 6.8 ns. In FBS, a bi-exponential decay suggested the presence of both aggregates and monomers. For PF, no effect of the concentration could be observed. In PBS, 8% of aggregates and 92% of monomers can be evaluated whereas it was 14–17% of aggregates and 83–86% of monomers in FBS.

### 2.4. Influence of the Temperature

**Figure 10:**
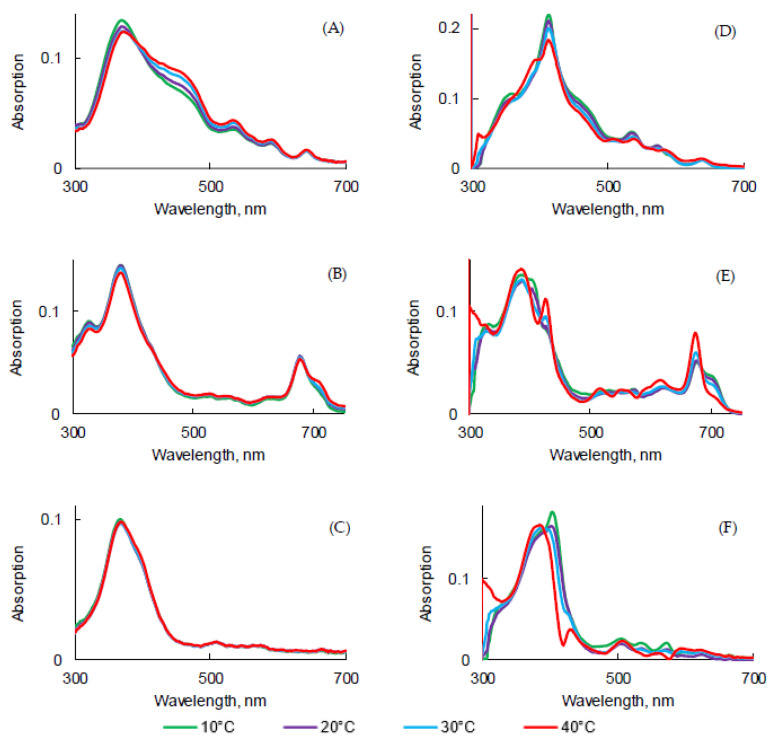
UV-visible absorption spectra (λ_exc_ = 400 nm for PpIX and PF and 415 nm for PPa) of PpIX (**A**,**D**), PPa (**B**,**E**), and PF (**C**,**F**) in PBS (**A**–**C**) and FBS (**D**–**E**) at different temperatures (*c* = 3.1 μM).

The influence of temperature on UV-visible absorption, fluorescence emission, and lifetime of PpIX, PPa, and PF in aqueous media was evaluated. UV-visible absorption and fluorescence emission spectra of the PSs in PBS and FBS after heating from 10 °C to 40 °C are presented in Figure 10.

For PpIX, in PBS, the intensity of the Soret band decreased with the increase of temperature (Figure 10A), whereas the intensity of the Q bands increased, except QI. In FBS (Figure 10D), a different behavior could be observed with a decrease of all band intensities, except QI. For PPa in PBS (Figure 10B), the intensity of the Soret band decreased with the increase of temperature and the QI band shape changed and was red-shifted from 680 nm to 712 nm with an isobestic point at 685 nm. In FBS (Figure 10E), the intensity of the Soret band increased with the increase of temperature as well as the QI band, with a change of shape and an isobestic point at the same 685 nm. For PF, almost no change could be observed in PBS (Figure 10C) whereas in FBS, a blue shift of the Soret band and a decrease of intensity was observed by increasing the temperature, as well as an increase of QI intensity (Figure 10F).

**Figure 11:**
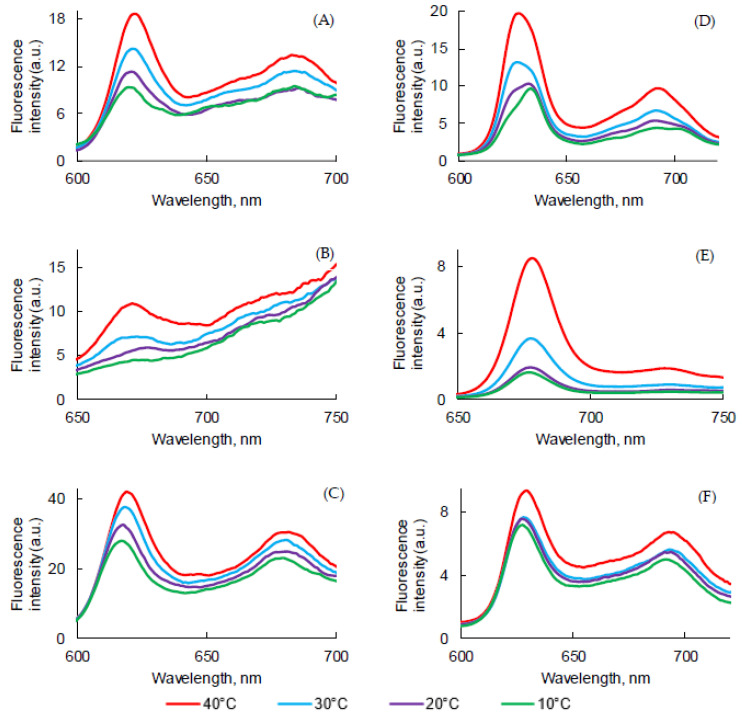
Fluorescence emission spectra (λ_exc_ = 400 nm for PpIX and PF and 415 nm for PPa) of PpIX (**A**,**D**), PPa (**B**,**E**), and PF (**C**,**F**) in PBS (**A**–**C**) and FBS (**D**–**F**) at different temperatures (*c* = 3.1 μM).

Fluorescence emission spectra were recorded in PBS and FBS at different temperatures (Figure 11). Whatever PS, the fluorescence emission intensity increased when the temperature rose from 10 to 40 °C. This might be have been because more monomers were in solution exhibiting fluorescence. For PpIX in PBS a slight red shift could be observed for the first band (Figure 11A) whereas in PBS, it was a slight blue-shift. No shift was detected for PPa (Figure 11B,E). Concerning PF, a red shift was observed both in PBS and FBS.

Fluorescence decays were recorded (Figure S3) and τ*_f_* were evaluated (Table 9). PpIX in PBS of FBS at different temperatures exhibited bi-exponential decay. The longest τ*_f_* likely corresponded to the monomer decay time and the shorter lifetime and was likely due to the aggregate decay time. A decrease of the shortest τ*_f_* could be observed with the increase of the temperature in both solutions. Moreover, the ratio aggregate/monomer also seemed to decrease with the increase of the temperature. For PPa, only one τ*_f_* was calculated in PBS. At low temperature (10 and 2 °C), both monomers and aggregates were present in FBS whereas aggregates disappeared at high temperature (30 and 40 °C). For PF, no effect of temperature was detected in PBS, whereas both short and long τ*_f_* decreased with temperature increase in FBS.

### 2.5. Influence of pH Medium

**Figure 12:**
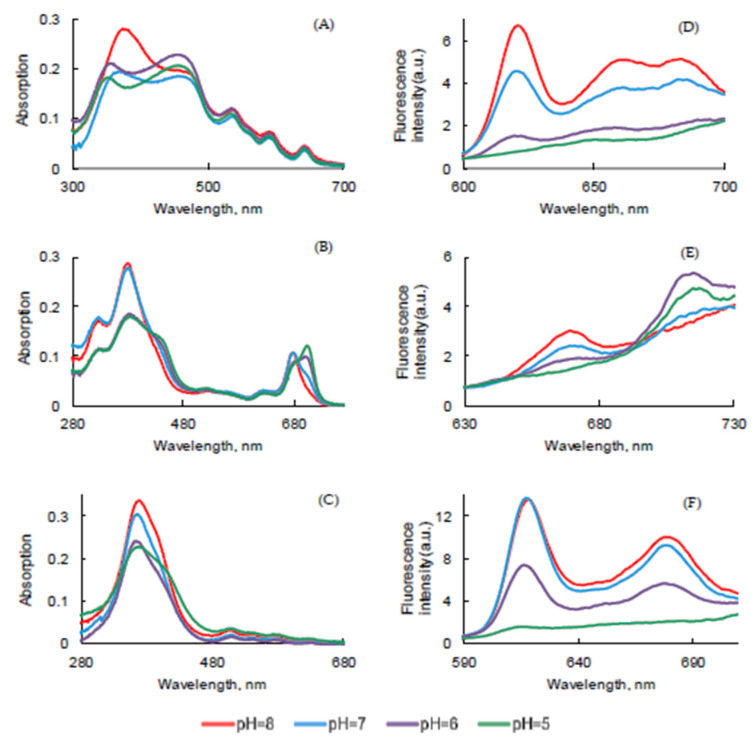
UV-visible absorption (**A**–**C**) and fluorescence emission (**D**–**F**) spectra (λ_exc_ = 400 nm for PpIX and PF and 415 nm for PPa) of PpIX (**A**,**D**), PPa (**B**,**E**), and PF (**C**,**F**) in PBS under different pH (*c* = 3.1 μM) at room temperature.

pH could also have an influence on photophysical properties. The UV-visible absorption, fluorescence emission, and lifetime of all PSs in PBS with a concentration of 3.1 μM were measured under different pH conditions (pH 5.0–8.0). For PpIX, increasing pH from 5 to 8 led to a red-shifted Soret band from 354 nm to 375 nm whereas the Q bands were pH-independent (Figure 12A).

By increasing the pH, PPa showed an increase of the Soret and QI band intensities (Figure 12B). Two isobestic points could be observed at 415 nm and 685 nm, exactly the same as those observed by changing the temperature. This is in good agreement with the presence of two different species that could be monomers or aggregates. The Soret band of PF increased with pH and the maximum of absorption and Q bands were not affected (Figure 12C).

For all PSs (Figure 12D–F), fluorescence increased with the increase of pH in relation to the formation of monomers and disappearance of aggregates [44,45,46] but we could also observe a decrease of the band at 717 nm for PPa (Figure 12E). Φ_f_ of all PSs in PBS under different pH medium were below 0.01.

*τ_f_* value of PpIX, PPa, and PF in PBS was also measured at different pH (Figure S4A). For PpIX, no aggregation could be observed at pH = 5 whereas aggregation occurred at pH 6–8 with the appearance of a short decay. At pH 5 and 6, the height of fast decay of PPa was higher than at pH 7 and 8, which was due to aggregation. At pH 5 and 6, the PPa was more aggregated with low *τ_f_* value, so only *τ_f_* of longer decay is given in the Table 10. For PF, *τ_f_* value of fast decay was around 3.0 ns and long decay 14.5 ns (Table 10).

## 3. Materials and Methods

Protoporphyrin IX, Pyropheophorbide-a, and Porfimer sodium (Photofrin^®^) were purchased from Sigma (Saint-Louis, MO, USA), BOC Sciences (Shirley, NY, USA), and Oncothai (Lille, France), respectively, and used without further purification. The stock solution of PpIX and PPa was prepared in dimethylsulfoxide (DMSO), and PF in methanol (MeOH). FBS was purchased from Sigma (Saint-Louis, MO, USA). PBS was prepared by mixing the exact volume of 0.2 M sodium phosphate, dibasic dehydrate and 0.2 M sodium phosphate, monobasic, monohydrate, and pH was adjusted to 7.4. The stock solution of SOSG in methanol was prepared by dissolving 100 µg vial in 33.0 µL of methanol and sodium azide solution was prepared in water with concentration of 0.15 M.

### 3.1. Spectroscopic Measurements

UV-visible absorption spectra were recorded on a UV-3600 UV-visible double beam spectrophotometer (Shimadzu, Marne La Vallee, France). Fluorescence spectra were recorded on a Fluorolog FL3-222 spectrofluorimeter (Horiba JobinYvon, Longjumeau, France) equipped with 450 W Xenon lamp, a thermo-stated cell compartment (25 °C), a UV-visible photomultiplier R928 (Hamamatsu, Japan) and an InGaAs infrared detector (DSS-16A020L Electro-Optical System Inc, Phoenixville, PA, USA). The excitation beam was diffracted by a double ruled grating SPEX monochromator (1200 grooves/mm blazed at 330 nm). The emission beam was diffracted by a double-ruled grating SPEX monochromator (1200 grooves/mm blazed at 500 nm). The ^1^O_2_ phosphorescence detection was measured with a HORIBA SpectraLED emitting at 415 nm, by a Multi-Channel Scaling (MCS) technique. The excitation pulse length was 102 µs and 600,000 pulses were averaged. ^1^O_2_ emission was detected through a double-ruled grating SPEX monochromator (600 grooves/mm blazed at 1 µm) and a long-wave pass (780 nm). All spectra were measured in 4-face quartz cuvettes. All the emission spectra (fluorescence and ^1^O_2_ luminescence) were displayed with the same absorbance (less than 0.2) with the lamp and photomultiplier correction.

Fluorescence quantum yield (Φ_f_) was calculated with tetraphenylporphyrin (TPP) in toluene as reference (Φ_f_ = 0.11) [47], using the following Equation (1):

(1) $$\Phi_{f}=\Phi_{f0}\times \frac{I_{f}}{I_{f0}}\times \frac{\mathrm{DO}}{{\mathrm{DO}}_{0}}\times {\left(\frac{n}{n_{0}}\right)}^{2}$$

where Φ_f_ and Φ_f__0_, I_f_ and I_f__0_, DO and DO_0_, and n and n_0_ are the quantum yields, fluorescence emission intensities, optical densities, and refraction indices of the sample and reference, respectively.

^1^O_2_ quantum yield (ΦΔ) was measured with TPP in toluene (ΦΔ = 0.68), rose Bengal in ethanol (EtOH) (ΦΔ = 0.68) and MeOH (ΦΔ = 0.76) as references [48,49] by Equation (2):

(2) $$\Phi_{\Delta }=\Phi_{\Delta 0}\times \frac{I_{f}}{I_{f0}}\times \frac{\mathrm{DO}}{{\mathrm{DO}}_{0}}$$

where Φ_Δ_ and Φ_Δ0_, I and I_0_, and DO and DO_0_ are the luminescence quantum yields of singlet oxygen, the luminescence intensities, and the optical densities of the sample and references, respectively.

### 3.2. Fluorescence and Luminescence Decays

Time-resolved experiments were performed using, for excitation, a pulsed laser diode emitting at 408 nm (LDH-P-C-400M, FWHM < 70 ps, 1 MHz) coupled with a driver PDL 800-D (both PicoQuant GmbH, Berlin, Germany) and for detection, an avalanche photodiode SPCM-AQR-15 (EG&G, Vaudreuil, QC, Canada) coupled with a 550 nm long-wave pass filter as detection system. The acquisition was performed by a PicoHarp 300 module with a 4-channel router PHR-800 (both PicoQuant GmbH, Berlin, Germany). The fluorescence decays were recorded using the single photon counting method. Data were collected up to 1000 counts accumulated in the maximum channel and analyzed using Time Correlated Single Photon Counting (TCSPC) software Fluofit (PicoQuant GmbH, Berlin, Germany) based on iterative deconvolution using a Levensberg–Marquandt algorithm. ^1^O_2_ lifetime (τ_Δ_) measurements were performed on a TEMPRO-01 spectrophotometer (Horiba Jobin Yvon, Palaiseau, France). The apparatus was composed of a pulsed diode excitation source SpectralLED-415 emitting at 415 nm, a cuvette compartment, a Seya–Namioka type emission monochromator (between 600 and 2000 nm) and a H10330-45 near-infrared photomultiplier tube with a thermoelectric cooler (Hamamatsu, Massy, France) for the detection. The system was monitored by a single-photon counting controller FluoroHub-B and the software DataStation and DAS6 (Horiba Jobin Yvon, Palaiseau, France).

## 4. Conclusions

This study focalized on three PSs that are used clinically (PpIX and PF) or for in vivo experiments (PPa). Our team proposed PPa coupled to folic acid to treat ovarian metastases by PDT (Patent WO/2019/016397).

By analyzing the photophysical properties of these three PSs in different conditions, we highlighted the fact that each PS is unique and reacts very differently depending on its chemical structure and concentration.

If the change of the medium polarity does not greatly affect the UV-visible absorption spectrum of PF, there is a drastic change for PpIX and PPa. In the literature, it is often claimed that PpIX should be excited at 630 nm in vitro or in vivo. This excitation wavelength is based on the absorption spectrum in ethanol. In FBS and PBS, which are aqueous media more similar to physiological media, the QI band is located at 641 nm.

Depending on the localization of the PS in the cells, the local viscosity can be very different. We could also observe that modifying the solvent viscosity did not greatly affect the maximal wavelengths of absorption of QI in PpIX and PF but it was blue-shifted for PPa for 10 nm (from 678 nm to 668 nm).

Temperature change slightly affected the UV-visible absorption spectra of PpIX and PF but drastically modified the UV-visible absorption of PPa in the range of 10 to 40 °C.

Finally, modifying pH also induced a shift of QI band for PPa of 25 nm (from 704 nm to 679 nm).

Perhaps the most interesting results are the Φ_Δ_ obtained in different solvents. Depending on the solvent, the values were totally different. In toluene, we could not detect any ^1^O_2_ whereas the Φ_Δ_ were quite good for PpIX and PPa 0.68 and 0.49, respectively. In EtOH, the Φ_Δ_ was 0.92, 0.53, and 0.80 for PpIX, PPa, and PF, respectively. If we switched to D_2_O, we could not detect any ^1^O_2_ of PpIX or PPa and the Φ_Δ_ was 0.15 for PF. Moreover, in real-life applications, the PS is ideally in a cellular context. The presence of protein, lipid, and other biomolecules molecules will also affect the photophysics of the PS. This raised the question of what type of experiments and which solvent should be used in the solution when performing in vitro studies.

## Figures and Tables

**Table 1:** Soret and Q bands (nm) of PpIX, PPa, and PF in different solvents at room temperature (*c* = 1.87 μM).

Solvent	ET(30)	PpIX					PPa					PF				
		Soret	QIV	QIII	QII	QI	Soret	QIV	QIII	QII	QI	Soret	QIV	QIII	QII	QI
Toluene	33.9	409	506	540	577	632	415	510	539	612	671	388	509	539	578	628
AcOEt	38.1	402	503	536	575	630	410	506	536	608	667	394	503	536	574	625
EtOH	51.9	402	503	537	575	629	411	509	539	609	667	398	503	536	574	625
MeOH	55.4	401	502	537	574	628	409	507	538	608	665	394	503	536	574	625
Glycerol	57.0	404	536	561	590	642	415	512	544	616	671	371	507	536	574	625
Water	63.1	352	532	557	589	641	380	522	554	625	677	365	507	542	567	616
PBS	≈63.1	365	532	557	589	641	379	526	558	630	677	365	507	542	567	616
FBS	-	409	506	537	585	640	405	515	546	617	675	391	506	538	573	624

**Table 2:** Molar extinction coefficient (ε, (M^−1^·cm^−1^)) of PpIX, PPa, and PF in different solvents at room temperature.

Solvent	PpIX					PPa					PF				
	Soret	QIV	QIII	QII	QI	Soret	QIV	QIII	QII	QI	Soret	QIV	QIII	QII	QI
Toluene	143,590	13,746	10,448	6481	5204	107,765	10,718	8589	7108	52,500	45,454	8535	5544	4705	2381
AcOEt	140,975	13,148	10,432	6113	5302	106,642	10,691	8455	6202	49,416	55,780	7351	4655	3854	2056
EtOH	142,440	13,305	10,615	6476	5067	92,874	9303	8403	7851	45,435	71,905	7832	4779	3661	1891
MeOH	139,797	12,410	10,048	6111	4579	94,334	9555	8771	8202	43,430	70,829	7333	4495	3497	1722
Glycerol	38,582	14,342	9180	8029	4909	46,057	5215	4989	4603	20,415	61,111	5151	3891	3531	1392
Water	40,603	15,326	9447	8177	5005	41,993	3900	3489	4179	17,311	61,617	3456	2806	2751	1044
PBS	47,433	11,565	7964	6945	4152	39,431	3699	3467	3937	15,444	59,023	3970	2786	2524	931
FBS	61,034	13,332	12,969	7436	4346	54,518	7172	6478	6808	31,092	45,952	5447	3515	2956	1387

**Table 3:** Φ_f_ of PpIX, PPa, and PF in different solvents at room temperature (*c* = 1.87 μM).

Solvent	ET(30)	Φ_f_ (± 0.01)		
		PpI	PPa	PF
Toluene	33.9	0.09	0.39	<0.01
AcOEt	38.1	0.06	0.34	<0.01
EtOH	51.9	0.08	0.39	0.07
MeOH	55.4	0.07	0.31	0.05
Glycerol	57.0	0.04	0.20	0.02
Water	63.1	<0.01	<0.01	0.01
PBS	≈63.1	<0.02	<0.01	0.01
FBS	-	<0.01	<0.01	<0.01

**Table 4:** Fluorescence lifetimes (τ*_f_*) of PpIX, PPa, and PF in different solvents at room temperature (*c* = 1.87 μM, λ_exc_ = 408 nm).

Solvent	τ*_f_* (ns)			
	PpIX	PPa	PF	References
Toluene	11.2 ± 0.06	6.7 ± 0.02	8.7 ± 0.2	This work
AcOEt	10.3 ± 0.1	6.6 ± 0.01	2.4 ± 0.2 (11%) 9.3 ± 0.2 (89%)	This work
EtOH	11.6 ± 0.1	6.6 ± 0.01	10.8 ± 0.1	This work
MeOH	11.0 ± 0.02	6.1 ± 0.01	10.2 ± 0.1	10.0 ± 0.6 [38] [PF]
Glycerol	2.9 ± 0.02 (66%) 6.4 ± 0.1 (34%)	6.5 ± 0.01	3.0 ± 0.01 (64%) 12.0 ± 0.1 (36%)	This work
Water	2.5 ± 0.05 (54%) 9.6 ± 0.4 (46%)	0.3 ± 0.02 (1%) 5.5 ± 0.1 (99%)	3.4 ± 0.08 (13%) 14.5 ± 0.08 (87%)	This work
PBS	3.0 ± 0.2 (27%) 13.0 ± 0.3 (73%)	1.4 ± 0.06 (15%) 5.7 ± 0.07 (85%)	2.2 ± 0.08 (12%) 14.1 ± 0.1 (88%)	13.2 ± 2.0 [38] [PF] 14.7 [39] [PF]
FBS	2.9 ± 0.2 (10%) 15.9 ± 0.3 (90%)	2.1 ± 0.2 (18%) 7.5 ± 0.1 (82%)	3.2 ± 0.2 (21%) 15.0 ± 0.2 (79%)	This work

**Table 5:** Φ_Δ_ of PpIX, PPa, and PF in different solvents at room temperature (*c* = 1.87 μM).

Solvent	Φ_Δ_ (± 0.10)		
	PpIX	PPa	PF
Toluene	0.68	0.49	0.01
EtOH	0.92	0.53	0.80
MeOH	0.92	0.42	0.61
D_2_O	-	-	0.15

**Table 6:** ^1^O_2_ lifetime of PpIX, PPa, and PF in different solvents at room temperature (*c* = 1.87 μM).

Solvent	τ_Δ_ (μs)			Literature Values
	PpIX	PPa	PF	
Toluene	30.4 ± 0.2	30.7 ± 0.2	-	30.5 ± 0.6 [42] [PS: 1H-phenalen-1-one-2- sulfonic acid (PNS)]
AcOEt	44.1 ± 0.6	43.2 ± 0.5	-	45 ± 1.5 [42] [PS: 1H-phenalen-1-one-2- sulfonic acid (PNS)]
EtOH	14.9 ± 0.6	14.6 ± 0.9	14.7 ± 0.8	15.3 ± 0.8, 12 [42,43] PS: hydrogen peroxide
MeOH	12.6 ± 0.9	8.9 ± 1.3	9.1 ± 1.0	9.9 ± 0.3, 7 [42,43] PS: ozone-triphenylphosphite

**Table 7:** Fluorescence lifetimes of PpIX, PPa, and PF (λ_exc_ = 408 nm, *c* = 3.1 μM) at room temperature.

(W/G, *v*/*v*)	τ*_f_* (ns)		
	PpIX	PPa	PF
W/G (100/0)	2.7 ± 0.02; 8.0 ± 0.1	0.3 ± 0.01; 5.5 ± 0.1	3.3 ± 0.1; 13.8 ± 0.2
W/G (80/20)	2.4 ± 0.04; 9.2 ± 0.3	0.1 ± 0.01; 5.6 ± 0.03	3.7 ± 0.02; 12.8 ± 0.04
W/G (60/40)	3.0 ± 0.01; 10.3 ± 0.1	0.1 ± 0.01; 5.7 ± 0.03	4.2 ± 0.02; 12.6 ± 0.04
W/G (40/60)	3.1 ± 0.02; 10.9 ± 0.2	5.5 ± 0.03	3.8 ± 0.04; 12.3 ± 0.1
W/G (20/80)	3.1 ± 0.05; 12.3 ± 0.4	5.9 ± 0.02	3.6 ± 0.02; 14.4 ± 0.05
W/G (0/100)	3.2 ± 0.02; 12.5 ± 0.2	6.5 ± 0.02	3.5 ± 0.01; 15.2 ± 0.05

**Table 8:** Fluorescence lifetimes of PpIX, PPa, and PF in PBS and FBS (λ_exc_ = 408 nm) at room temperature.

Concentration (μM)	τ*_f_* (ns)		
	PpIX	PPa	PF
**PBS**			
2.1	3.8 ± 0.2 (21%) 14.5 ± 0.2 (79%)	6.8 ± 0.1	2.1 ± 0.3 (8%) 14.0 ± 0.2 (92%)
4.2	3.6 ± 0.2 (22%) 13.8 ± 0.2 (78%)	5.9 ± 0.1	2.7 ± 0.4 (8%) 14.8 ± 0.2 (92%)
6.3	3.5 ± 0.2 (25%) 12.8 ± 0.2 (75%)	5.8 ± 0.1	2.4 ± 0.3 (8%) 14.7 ± 0.2 (92%)
8.4	3.8 ± 0.2 (27%) 13.7 ± 0.2 (73%)	5.6 ± 0.1	2.6 ± 0.4 (8%) 14.8 ± 0.2 (92%)
**FBS**			
2.1	3.7 ± 0.2 (13%) 17.0 ± 0.1 (87%)	3.5 ± 0.3 (13%) 8.2 ± 0.1 (87%)	3.3 ± 0.3 (17%) 15.3 ± 0.2 (83%)
4.2	3.6 ± 0.3 (12%) 17.1 ± 0.2 (88%)	2.5 ± 0.6 (5%) 8.2 ± 0.1 (95%)	3.6 ± 0.3 (15%) 15.5 ± 0.2 (85%)
6.3	3.9 ± 0.4 (11%) 17.8 ± 0.2 (89%)	2.6 ± 0.5 (5%) 8.2 ± 0.1 (95%)	3.4 ± 0.3 (14%) 15.6 ± 0.2 (86%)
8.4	3.9 ± 0.4 (11%) 17.8 ± 0.2 (89%)	3.2 ± 0.2 (4%) 8.5 ± 0.2 (96%)	3.4 ± 0.3 (14%) 15.7 ± 0.2 (86%)

**Table 9:** Fluorescence lifetimes of PpIX, PPa, and PF (λ_exc_ = 408 nm, *c* = 3.1 μM).

Temperature, °C	Fluorescence Lifetime (ns)		
	PpIX	PPa	PF
**PBS**			
10	5.7 ± 0.4 (19%) 16.4 ± 0.3 (81%)	5.8 ± 0.1	2.4 ± 0.3 (9%) 14.6 ± 0.2 (91%)
20	4.6 ± 0.3 (16%) 15.4 ± 0.2 (84%)	5.6 ± 0.1	2.9 ± 0.3 (9%) 14.8 ± 0.2 (91%)
30	4.6 ± 0.4 (13%) 13.9 ± 0.2 (87%)	5.5 ± 0.1	2.6 ± 0.2 (9%) 14.6 ± 0.2 (91%)
40	4.0 ± 0.4 (13%) 14.1 ± 0.2 (87%)	5.3 ± 0.1	2.6 ± 0.3 (9%) 14.6 ± 0.2 (91%)
**FBS**			
10	3.9 ± 0.5 (9%) 18.0 ± 0.2 (91%)	2.8 ± 0.3 (10%) 7.8 ± 0.1 (90%)	3.0 ± 0.2 (16%) 15.0 ± 0.2 (84%)
20	3.4 ± 0.4 (8%) 18.0 ± 0.2 (92%)	2.0 ± 0.3 (7%) 7.7 ± 0.1 (93%)	2.8 ± 0.2 (16%) 14.7 ± 0.2 (84%)
30	3.3 ± 0.4 (7%) 17.9 ± 0.2 (93%)	7.3 ± 0.1	2.8 ± 0.2 (16%) 14.7 ± 0.2 (84%)
40	3.2 ± 0.4 (6%) 17.6 ± 0.2 (94%)	7.2 ± 0.1	2.6 ± 0.2 (16%) 13.8 ± 0.2 (84%)

**Table 10:** Fluorescence lifetimes of PpIX, PPa, and PF in PBS (λ_exc_ = 408 nm, *c* = 3.1 μM) at room temperature.

pH	τ*_f_* (ns)		
	PpIX	PPa	PF
5	5.0 ± 0.2	3.5 ± 0.4	2.2 ± 0.2 (21%) 11.2 ± 0.2 (79%)
6	5.7 ± 0.4 (42%) 13.1 ± 0.5 (58%)	5.1 ± 0.3	3.6 ± 0.5 (9%) 14.8 ± 0.2 (91%)
7	4.3 ± 0.06 (30%) 14.6 ± 0.1 (70%)	5.5 ± 0.1	2.7 ± 0.3 (9%) 15.0 ± 0.2 (91%)
8	3.7 ± 0.2 (23%) 14.6 ± 0.2 (77%)	5.6 ± 0.09	2.4 ± 0.2 (8%) 14.4 ± 0.2 (92%)

## Data Availability

The data presented in this study are available on request from the corresponding author.

## Supplementary Materials

The following are available online at https://www.mdpi.com/1424-8247/14/2/138/s1, Figure S1: Fluorescence decay of PpIX (A), PPa (B), and PF (C) in water/glycerol mixture (λ_exc_ = 408 nm, *c* = 3.1 μM). Figure S2: Fluorescence decay of PpIX (A), PPa (B), and PF (C) in PBS at different concentrations (λ_exc_ = 408 nm). Figure S3: Fluorescence decay of PpIX (A), PPa (B), and PF (C) in PBS at different temperatures (λ_exc_ = 408 nm, *c* = 3.1 μM). Figure S4: Fluorescence decay of PpIX (A), PPa (B), and PF (C) in PBS under different pH (λ_exc_ = 408 nm, *c* = 3.1 μM). Table S1: Fluorescence emission bands (nm) of PpIX, PPa, and PF in different solvents (*c* =1.87 μM).
