# Supplementary material for: Photophysical Properties of Protoporphyrin IX, Pyropheophorbide-a, and Photofrin® in Different Conditions
Source: Pharmaceuticals (Basel). 2021 Feb 9;14(2):138. doi: 10.3390/ph14020138 (PMC7914864; doi:10.3390/ph14020138)
Supplement: Supplementary file 1 [file pharmaceuticals-14-00138-s001.pdf]

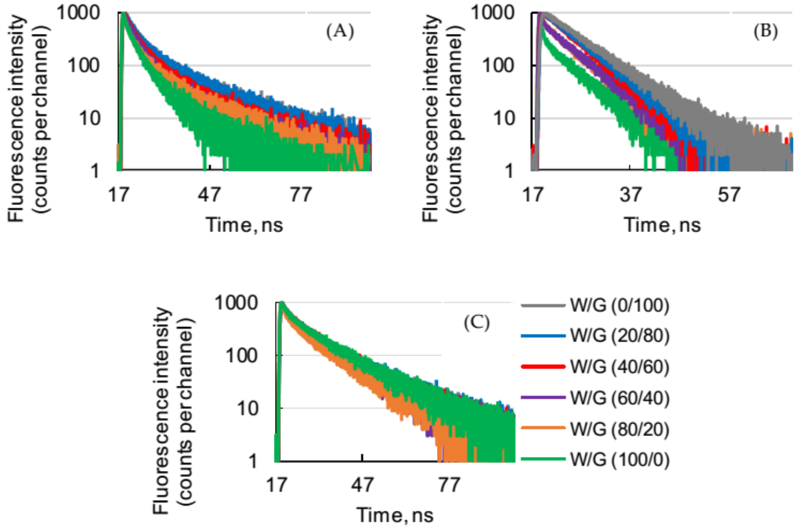

Figure S1. Fluorescence decay of PpIX (A), PPa (B) and PF (C) in water/glycerol mixture (λ_{exc} = 408 nm, C = 3.1 μM).

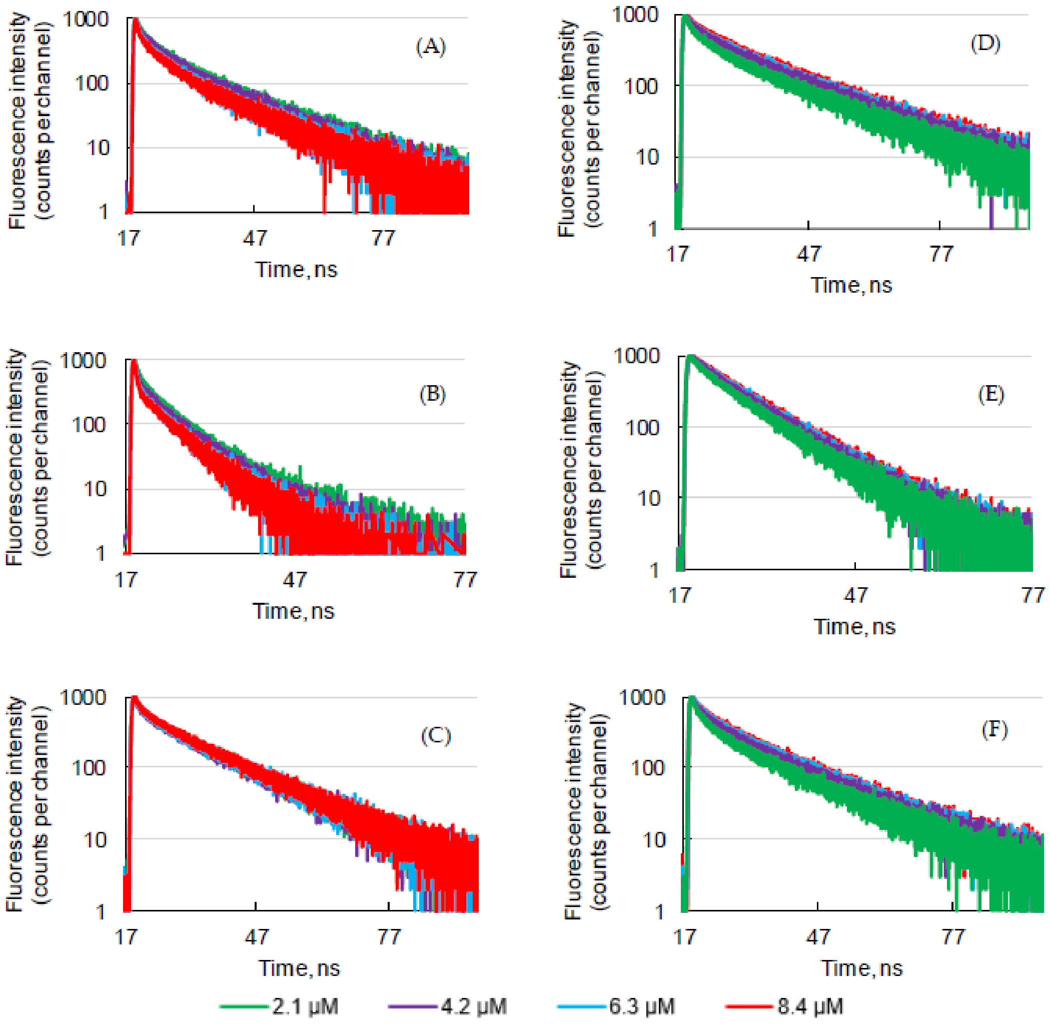

Figure S2. Fluorescence decay of PpIX (A, D), PPa (B, E), PF (C, F) in PBS and FBS at different concentrations (λ_{exc} = 408 nm).

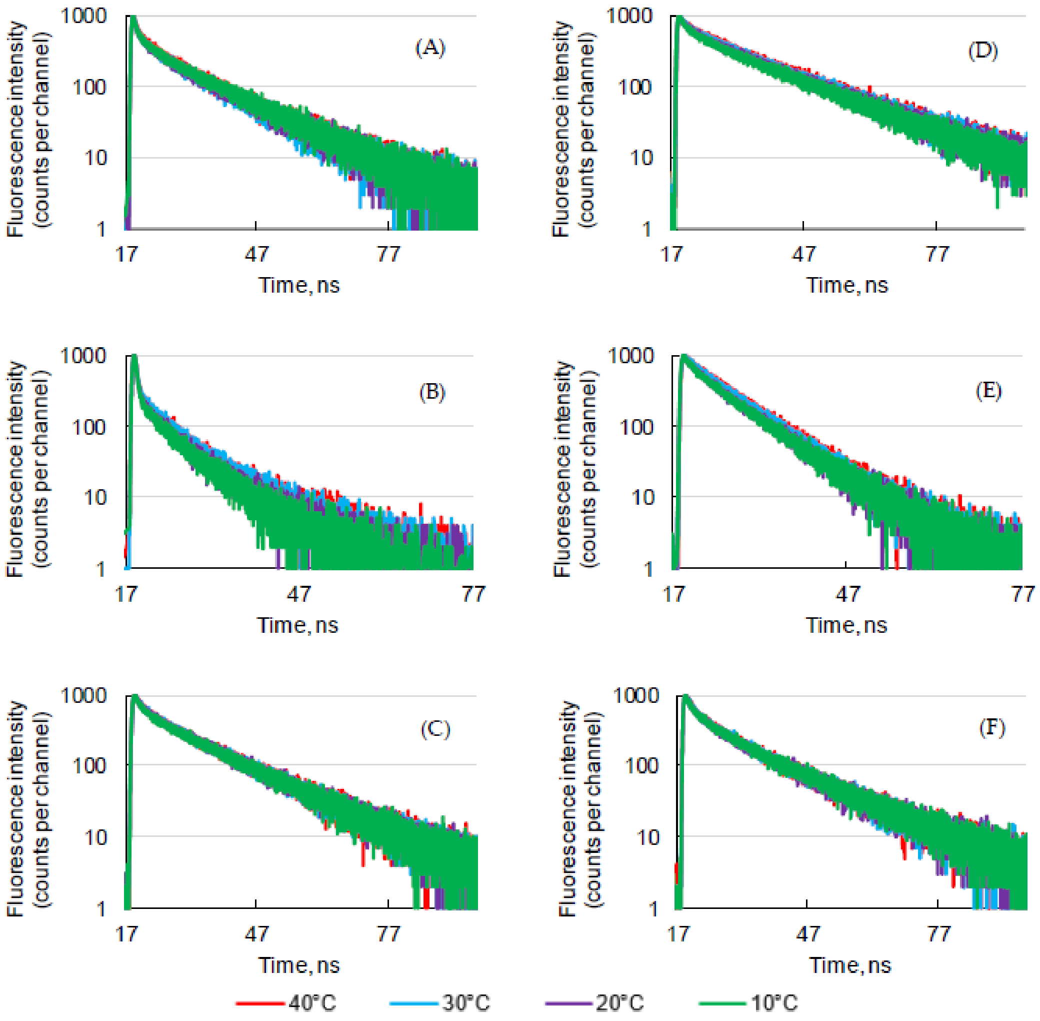

Figure S3. Fluorescence decay of PpIX (A, D), PPa (B, E), PF (C, F) in PBS and FBS at different temperatures (λ_{exc} = 408 nm, C = 3.1 μM).

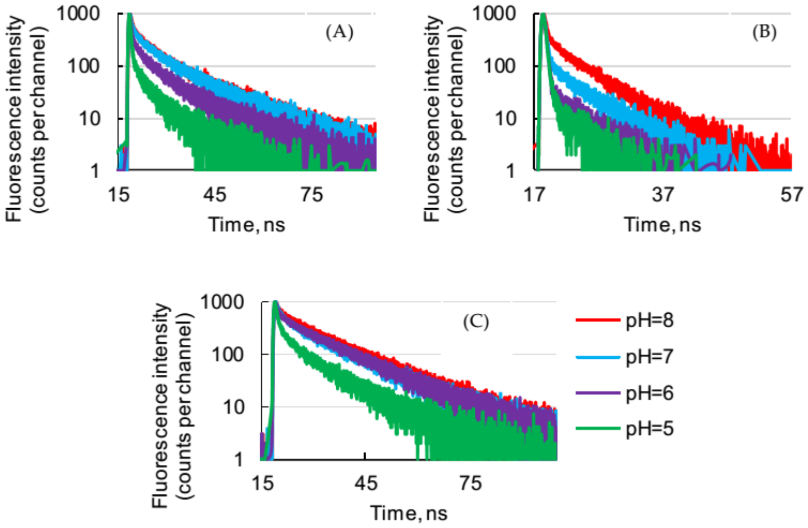

Figure S4. Fluorescence decay of PpIX (A), PPa (B) and PF (C) in PBS under different pH (λ_{exc} = 408 nm, C = 3.1 μM).

Table S1. Fluorescence emission bands (nm) of PpIX, PPa and PF in different solvents (C=1.87 μM).

Solvent	Protoporphyrin IX (λ_{exc} = 400 nm)		Pyropheophorbide a (λ_{exc} = 415 nm)		Photofrin (λ_{exc} = 400 nm)	
	**1 band**	**2 band**	**1 band**	**2 band**	**1 band**	**2 band**
Toluene	635	704	675	722	633	696
AcOEt	632	701	672	718	629	694
EtOH	632	701	673	719	629	694
MeOH	631	698	672	719	628	693
Glycerol	630	693	675	723	625	690
Water	622	685	672	719	617	678
PBS	621	686	672	719	617	678
FBS	625	691	678	727	628	695
